# The efficacy of hydrogel containing zinc oxide-loaded and minocycline serum albumin nanopartical in the treatment of peri-implantitis

**DOI:** 10.4317/medoral.25890

**Published:** 2023-07-10

**Authors:** Xingjia Li, Changyong Yuan, Qixin Chen, Qiang Xue, Jie Mou, Penglai Wang

**Affiliations:** 1 MDS. Department of Prosthodontics, Wuxi Stomatology Hospital, Wuxi, China; 2 PhD. Department of Oral Implantology, Affiliated Stomatological Hospital of Xuzhou Medical University, Xuzhou, China; 3 MDS. Department of Oral Implantology, Affiliated Stomatological Hospital of Xuzhou Medical University, Xuzhou, China; 4 PhD. School of Pharmacy, Xuzhou Medical University, Xuzhou, China

## Abstract

**Background:**

We conducted this animal study to assess the efficacy of the novel hydrogel containing zinc oxide-loaded and minocycline serum albumin nanoparticals (Mino-ZnO@Alb NPs) on peri-implantitis in an experimental mouse model.

**Material and Methods:**

Mino-ZnO@Alb NPs was prepared as previously reported. The peri-implantitis model was successfully established in rats, and the rats were divided into three groups randomly: Mino-ZnO@Alb NPs (Mino-ZnO) group, minocycline group, and untreated group. Four weeks later, clinical and radiographic assessments were performed to evaluate soft tissue inflammation and bone resorption level. Histologic analysis was performed to estimate the amount of remaining supporting bone tissue (SBT) around implants. ELISA tests were used to determine the concentration of inflammation factor interleukin-1-beta (IL-1β) and anti-inflammation factor tumor necrosis factor-alpha (TNF-α) around implants.

**Results:**

After one month, the Mino-ZnO group showed better results than the other two groups in regards to the results of bleeding on probing, probing pocket depth, bleeding index and gingival index. X-ray showed that SBT at mesial and distal sites around implants in the other two groups was significantly lower compared with that of Mino-ZnO group. The quantity of osteoclasts in peri-implant tissues of the Mino-ZnO group was less than that in the minocycline and untreated groups. IL-1β in the Mino-ZnO group was lower than that in the other two groups. TNF-α level was the opposite.

**Conclusions:**

Mino-ZnO@Alb NPs can effectively treat peri-implantitis and promote soft tissue healing, and may act as a promising product.

** Key words:**Minocycline, zinc oxide, nanohydrogel, peri-implantitis, rats.

## Introduction

Peri-implantitis is a common complication that can occur after dental implantation, resulting in the gradual loss of soft and hard tissues, and even implant failures (1). The cause of peri-implantitis, including bacterial biofilms, insufficient primary stability during implant placement, occlusal trauma, pain, neuropathy and smoking (2). The widely accepted cause is bacterial biofilm (3). It is a major reason inducing peri-implantitis. Therefore, treatment of peri-implantitis aims to reduce the total peri-implant bacterial load.

Various methods have been attempted to eliminate bacterial biofilm and treat peri-implantitis, including mechanical debridement and drug therapy. Mechanical debridement alone is not always effective, as it does not completely eliminate clinical signs of the condition (4,5). Other approaches such as laser therapy and photodynamic therapy have shown moderate to little effects on peri-implantitis lesions (6,7). Conventional drug treatments, such as topical minocycline and metronidazole, have limited therapeutic effects, short residence times, and low predictability (8,9). In this study, we used a novel drug that is a hydrogel (referred to as Mino-ZnO@Alb NPs) to treat peri-implantits (10). This drug containing zinc oxide nanoparticles and minocycline were prepared. It has been reported that several kinds of bacterials, such as *S. mutans* and S. anginosus, which play an important role in inducing peri-implantitis (11). According to our previous study, this drug, namely, Mino-ZnO@Alb NPs, was effective in killing these bacteria. This drug has many superior characteristics and can make up for the shortcomings of traditional drugs. It has many powerful functional properties, including broad antibacterial spectrum, long-term sustained release, tissue repair and adhesion properties. Compare with other therapy could be a better way to treatment peri-imolantitis as it can increase bioavailability and the residence time around the implant (10).

This study aims to evalute the effectiveness of Mino-ZnO@Alb NPs for treating peri-implantitis compared with the conventional drug minocycline.

## Material and Methods

This project was examined and verified by the Laboratory Animal Ethics Committee at Xuzhou Medinical University in accordance with the Cuide to Laboratory Animal Ethics Examination by Xuzhou Medinical University and Use Committee (201909w009). The experimental study was conducted from September 2019 until June 2020. This article was written according to the ARRIVE guidelines (12).

- The preparation of Mino-ZnO@Alb NPs hydrogels

This drug-loaded nanopartical including zinc oxide, albumin, and minocycline was known as Mino-ZnO@Alb NPs. The hydrogels containing Mino-ZnO@Alb NPs were prepared as previously reported (10). In short, the modified reversed-phase microemulsion technique was employed to produce ZnO nanoparticles, with the zinc acetate serving as the source material. By means of the emulsification cross-linking method, the albumin microspheres were fabricated. To produce Mino-ZnO@Alb NPs HG, a dispersion of Carbopol 940VR (a type of acrylic acid polymer) was blended with the Mino-ZnO@Alb NPs nanoparticle solution at a concentration of 1% (w/w).

- Establishment of peri-implantitis rat model

18 Sprague-Dawley (SD) rats (male, 4 weeks, 150 ± 20g) purchased from Xuzhou Medinical University (Xuzhou, China) were housed in standard cages. All animals can move freely during the entire experiment.

- Tooth extraction and implant placement

For anesthetizing, chloral hydrate (10%, 0.3 mL/100g, Sigma-Aldrich, St. Louis, MO, USA) was used and rats were fixed on the sterile Table. The maxillary first molars of these rats were extracted. One month after healing, custom-made, pure titanium implants (2 mm in diameter and 4 mm in length) were obtained commercially (WEGO JERICOM Biomaterials Co., Ltd, Shandong, China), and implanted (Supplement 1).

- Induction of peri-implantitis

Implant osseointegration was assessed after 4 weeks. 5-0 silk ligatures (Ethicon, Inc., New Jersey, USA) were used for inducing peri-implantitis (Supplement 1) (13). The rats were fed with 10% of sugar water and soft food.

- Treatment of peri-implantitis

Peri-implantitis was induced successfully after 2 weeks. The rats were divided into (6 per group) Mino-ZnO group (Jiangsu Key Laboratory of New Drug and Clinical Pharmacy, Xuzhou Medinical University, Xuzhou, China), minocycline group (Perio®, Sunstar INC, Osaka, Japan) and untreated group randomly. The Mino-ZnO@Alb NPs hydrogels (Mino-ZnO group) and minocycline (minocycline group) were injected once a week for two weeks around the neck of the implant where the peri-implantitis occurred.

- Peri-implant soft tissue inflammatory parameters

Bleeding on probing (BOP), probing pocket depth (PPD), bleeding index (BI) and gingival index (GI) were collected before and post treatment after 4 weeks. PPD was measured at four sites per implant (mesial buccal and lingual, distal and lingual).

- Tissue collection and sample preparation

The rats were euthanized (Supplement 1), and the maxillas were then removed and hemisected. These samples were fixed in 4% paraformaldehyde (PFA, Sigma-Aldrich) for 48 h. Then they were decalcified in 10% ethylenediaminetetraacetic acid (EDTA, Sigma-Aldrich) for one month in 4°C with shaking. For immunohistological analysis, all the maxillas were cut in 3-µm sections. The gingival tissues around implants from rats were collected and stored in -80°C for protein determination.

- Imaging analysis for supports bone tissue

Support of bone tissue (SBT) and peri-implantitis development were corroborated by radiographic x-rays of the rats. Each implant measured mesial and distal site. All radiographs were measured by a software to analyze images (Image J, National Institute of Health, Bethesda, MD, USA). All images were calibrated using a known length (length of the implant).

- Histological analysis

Hematoxylin and eosin (H&E) stain

Maxillary slices were stained with hematoxylin and eosin and observed using a inverted routine microscope (Nikon, Tokyo, Japan) for histological analysis. Inflammatory cells and bone morphology surrounding the implant were observed at magnification of 40×. on each section.

Tartrate-resistant acid phosphatase (TRAP) staining

TRAP staining used an acid phosphatase kit (Servicebio, Wuhan, China). After staining for 30 minutes and hematoxylin counterstaining for 1 minute, the osteoclasts we calculated are cells with three or more nuclei as previously described (14). In samples of peri-implantitis, the region of interest (ROI) is defined as a 2×4 mm rectangular area. Each slice was observed at magnification of 10×. The number of osteoclasts in the ROI was manually quantified by Image-J.

- Enzymatic immunosorbent assay (ELISA)

Measurement of interleukin-1-beta (IL-1β) and tumor necrosis factor-alpha (TNF-α)

Gingival tissues were obtained around the implants and used for extracting protein. Aliquots of each sample were tested using ELISA to determine the concentration of IL-1β (IL1-beta ELISA Kit, Protenitech, Chicago, IL, USA) and TNF-α (Rat TNF-alpha ELISA Kit, Protenitech). Dilute the antibody with carbonate coating buffer to a protein content of 1-10 μg/mL. Add 100 μL to each well of the polystyrene microplate, overnight at 4°C. The next day, discard the solution in the well and wash with washing buffer 3 times for 3 minutes each time. Add 200 μL of closed solution to each hole and incubate at 37 ° C for 2 hours. Carefully remove the sealing film, put it in the washing machine and clean it 3-5 times. 100 μL of properly diluted test sample is added to the reaction hole of the above package. Seal the plate with a sealing film and incubate for 1-2 hours at 37°C. Wash, add 100 μL diluted biotin antibody working solution to each well. Incubate the sealing plate at 37°C for 1 hour after sealing the plate with the sealing film. Wash and add 100 μL of diluted enzyme binding working solution to each well. After sealing the plate with the sealing film, incubate at 37°C in the dark for 30 minutes. Wash, add 100 μL substrate solution to each hole and react for 10-30 minutes in darkness at 37°C until a clear color gradient appears in the multiple dilution hole. Add 100 μL sulfuric acid to each reaction hole to terminate the reaction, and the color changes from blue to yellow. Result determination: Within 10 minutes, measure the OD value of each well after zeroing the blank control well at 450 nm on the microplate reader. (BioTeK/ Epoch, Vermont, USA).

- Statistical analysis

The outcomes use the mean ± standard deviation (SD). The primary outcome was PPD, BI, GI, SBT, osteoclast numbers, the concentration of IL-1β and TNF-α. Mean values and SD of all parameters were calculated using a common software (SPSS 22.0, SPSS Inc., Chicago, USA). The error of α is 0.05. Normal distribution was tested by Shapiro Wilk test. In order to compare the differences between groups post treatment (after 4 weeks), all data were analyzed by one-way ANOVA and Chi-square’s test for BOP.

Resuits

- Mino-ZnO@Alb NPs curbed peri-implant soft tissue inflammatory reaction

**Figure 1:**
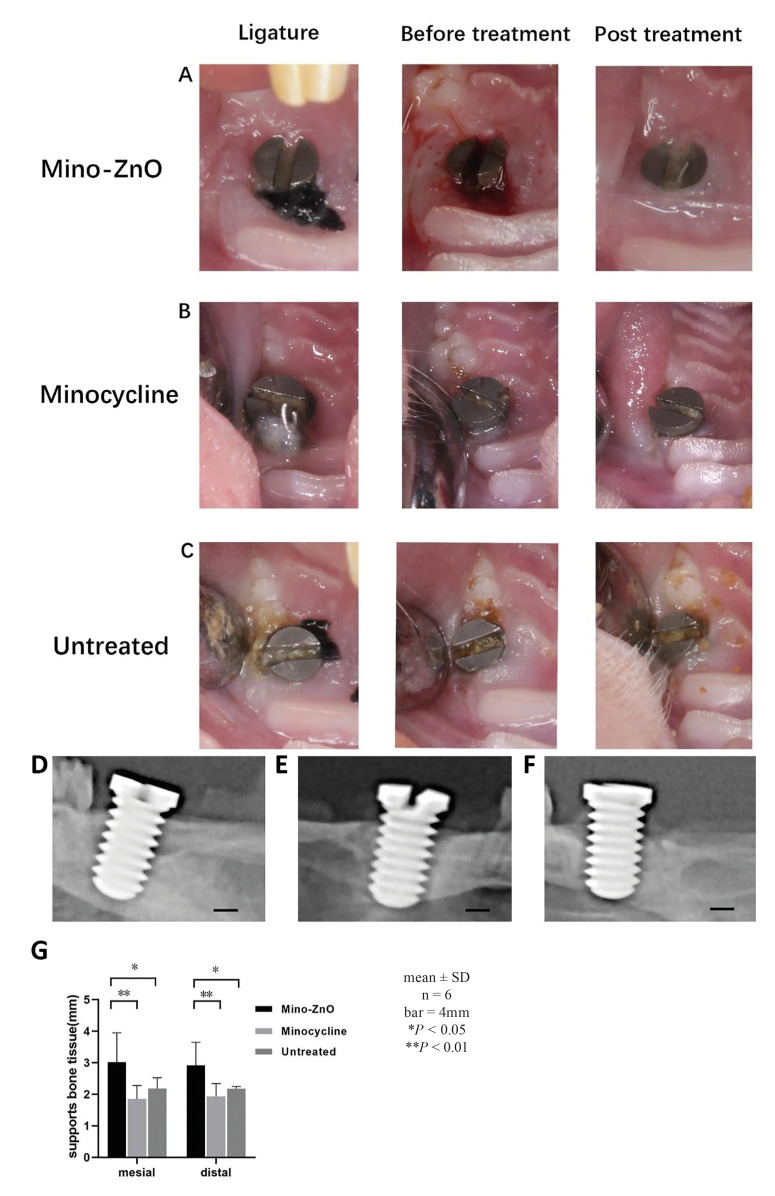
Representative photographs of implant and soft tissue condition at the time of ligature, before and 4-weeks post treatment and radiographic evaluation of SBT after 4 weeks of treatment. (A) Mino-ZnO group, (B) Minocycline group, (C) Untreated group. (D) Mino-ZnO group, (E Minocycline group, (F) Untreated group. (G) The level of SBT was measured.

Overall, 30 implants of 18 rats achieved osteointegration (no mobility and bleeding upon touching and probing). The soft tissue around the implant recovered healthy after 4 weeks of treatment in Mino-ZnO group (Fig. 1). There were significant differences in PPD (DB and DL), BI, GI and BOP between the Mino-ZnO and Minocycline group (DB: *P* = 0.021, DL: *P* = 0.001, BI: *P*= 0.046, GI: *P* = 0.046, BOP: *P* = 0.042, Table 1). The differences of PPD (DB and DL), BI, GI and BOP between Mino-ZnO group and Untreated group were also statistical significant (DB: *P*= 0.027, DL: *P* = 0.001, BI: *P* = 0.003, GI: *P* = 0.003, BOP: *P* = 0.005, Table 1). Additionally, there were more bleeding sites in the untreated group comparing with the group of Mino-ZnO (*P* < 0.05, Fig. 1). There was no statistical difference of these parameters between Minocycline group and Untreated group (*P* > 0.05, Table 1). In the Mino-ZnO group, 90.9% (10 of 11) of implants achieved a successful therapeutic effect (improvement of BOP, Bi, GI and shallow periodontal pocket), whereas only 72.7% (8 of 11) and 50% (4 of 8) of implants appeared positive results in the Minocycline and Untreated group, respectively. These results indicated that Mino-ZnO@Alb NPs had stronger capabilities to promote soft tissue healing by reducing inflammation in patients with peri-implantitis.

**Table 1:**
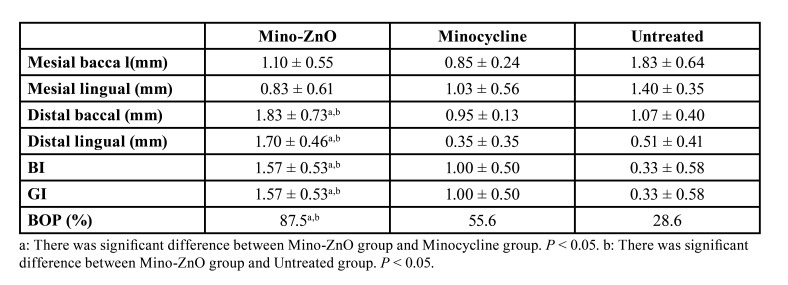
PPD, BI and GI before and after treatment (Mean ± SD).

- Mino-ZnO@Alb NPs restrained peri-implant bone resorption

X-ray analysis appears that there was lower bone resorption in the Mino-ZnO group (Fig. 1) after 4 weeks of treatment compared with the minocycline group (Mesial: *P* = 0.001, Distal: *P* = 0.002, *P* < 0.05, Table 2) and untreated group (Mesial: *P* = 0.036, Distal: *P* = 0.038, *P* < 0.05, Table 2). There was no statistical difference of SBT between the minocycline and untreated group (*P*>0.05, Fig. 1). In the Mino-ZnO group 72.7% (8 of 11) of implants displayed no further bone loss; this was only 63.6% (7 of 11) in the Minocycline group and 62.5% (5 of 8) in the untreated group. These consequences exposed that Mino-ZnO@Alb NPs could promote soft tissue healing by decreasing bone resorption in patients with peri-implantitis.

- Mino-ZnO@Alb NPs inhibited peri-implant neutrophil infiltration and reduced the number of osteoclasts

**Figure 2:**
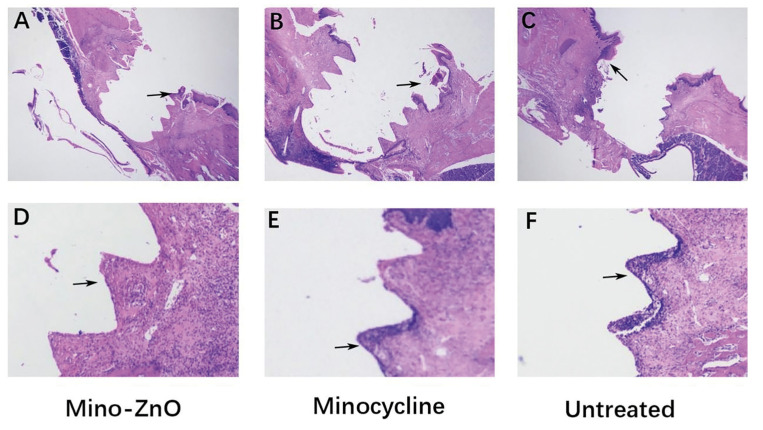
H&E staining of peri-implant tissues in Mino-ZnO (A, D) Minocycline (B, E) and Untreated (C, F) group. (A- C 10X magnification, D- F, 40X magnification).

**Figure 3:**
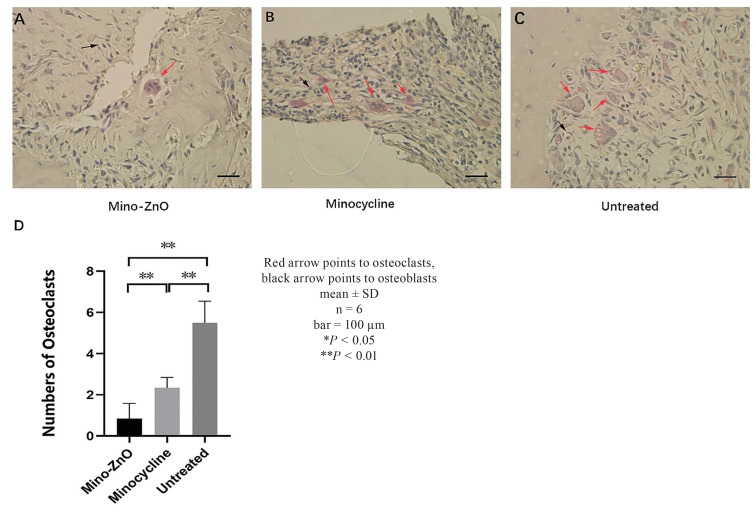
Osteoclasts after after 4 weeks of treatment for peri-implantitis. TRAP staining of samples in the Mino-ZnO (A), Minocycline (B) and Untreated (C) group after treatment (10X magnification). (D) The number of osteoclasts was measured.

In this part, we carried out histological analysis. The result of H&E stain assay exposed that compared with the Mino-ZnO group, the Minocycline and Untreated group showed obvious neutrophil infiltration. The bone contact area in the Minocycline and the untreated group was significantly reduced, and the bone in several threaded sites was absorbed and replaced by inflammatory granulation tissue (Fig. 2). In the Mino-ZnO group, the contour of the implant in the bone was clear-cut (Fig. 2). The results of trap stain assay displayed that higher numbers of osteoclasts were counted in the untreated group (Fig. 3). The numbers of osteoclasts were lowest in the Mino-ZnO group compared with those in the Minocycline g (*P* = 0.006, *P* < 0.01, Fig. 3) and Untreated group (*P* = 0.001, *P* < 0.01, Fig. 3). Hence, we confirmed that Mino-ZnO@Alb NPs could promote soft tissue healing by decreasing neutrophil infiltration and reducing the number of osteoclasts in patients with peri-implantitis.

**Table 2:**
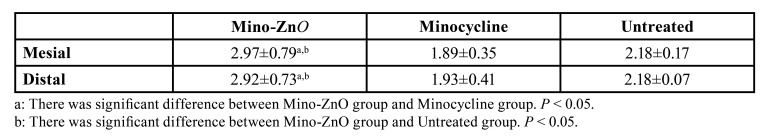
Radiographic measurements of (Mean values ± SD, mm) after 4 weeks treatment.

**Figure 4:**
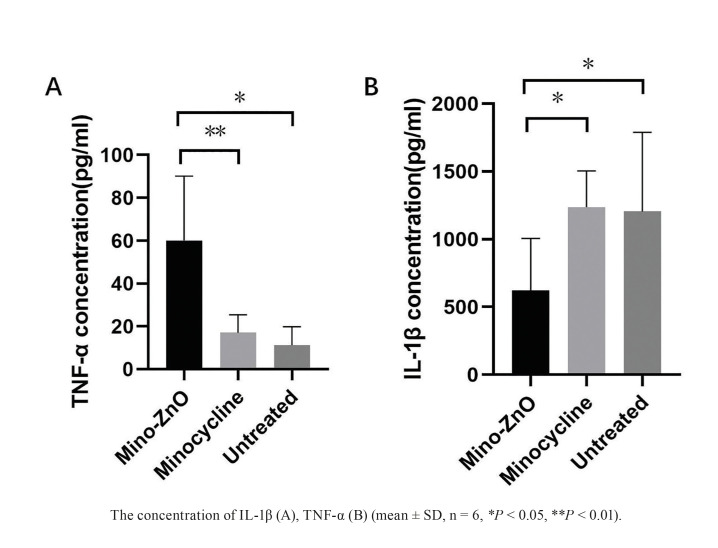
Inflammatory and anti-inflammatory cytokines in the Mino-ZnO, Minocycline and Untreated group after 4 weeks treatment.

- Mino-ZnO@Alb NPs reduced IL-1β level but enhanced the TNF-α level in peri-implant gingiva

In this part, we carried out ELISA assay to measure the levels of IL-1β and TNF-α in peri-implant gingiva. For IL-1β, there was a statistically significantly lower level of expression in the Mino-ZnO group than that in the minocycline group (*P* = 0.003, *P* < 0.01, Fig. 4) and the untreated group (*P* = 0.046, *P* <0.05, Fig. 4). While TNF-α, there was a significant upregulation in the Mino-ZnO group compared with the minocycline group (*P* = 0.028, *P* < 0.05, Fig. 4) and untreated group (*P* = 0.047, *P* < 0.05, Fig. 4). Moreover, there was no statistical difference in IL-1β and TNF-α expression between the Minocycline and Untreated group (*P* > 0.05, Fig. 4). We revealed that Mino-ZnO@Alb NPs could reduce IL-1β level but enhance the TNF-α level in peri-implant gingiva to promote soft tissue healing in patients with peri-implantitis.

## Discussion

Peri-implantitis is a complex issue that puzzles doctors and patients. Compared with minocycline, a traditional medicine for treating peri-implantitis, the new drug Mino-ZnO@Alb NPs has greater advantages and better effect in treatmenting peri-implantitis (15-18). It can be seen that with the extension of the treatment time, the PPD, BI, GI and BOP of the Mino-ZnO group and the minocycline group all decreased, but these indexes in the Mino-ZnO group decreased significantly more (Table 1). This indicated that Mino-ZnO@Alb NPs could reduce the inflammation and restore the soft tissue to health around implants. However, if the degree of inflammation is severe and the implant relaxes twice or three times, the PPD will not decrease, but it will continue to deepen (19). The results of H&E staining and trap staining explained that the number of osteoclasts (20-22) in the Mino-ZnO group was less than the minocycline and untreated group (Fig. 1, Fig. 2). The bone tissue was not damaged severely around the implant (Fig. 1). X-ray results appeared that the loss of bone in the Mino-ZnO group was less than that in the other two groups (23). This result was also consistent with the Trap staining outcome (24). From the results of the ELISA, the anti-inflammatory factor TNF-α in the Mino-ZnO group was more than the other two groups (Fig. 4) and the inflammatory factor IL-1β was less than the other two groups (Fig. 4). This demonstrates that this medicine has good anti-inflammatory effects.

Previous studies have reported that Mino-ZnO@Alb NPs could inhibit several common pathogens in the inflammation tissue around the implant, such as *Streptococcus mutans* and *Streptococcus* vasospasm (10). However, the mechanism is unclear. It is assumed that the drug may affect the inflammatory mechanisms (25-27) around the implant or affect the structure of these characteristic flora (28). We can construct a scaffold material to direct drugs to different pathogenic structures of bacteria. Exploring which structure of the bacteria this drug has a destructive effect on may be a more targeted way to inhibit bacteria, and eliminate inflammation (29). In this way, the curative effect may be improved, the treatment cycle will be greatly shortened, and the patient's pain will be reduced. There are still some limitations in this paper, and there are still some differences between the animal model used in this study and the actual situation. Peri-implantitis can occur in any stage of dental implant treatment, and in many cases, it happens after the crown placement. While in our study, the peri-implantitis model didn’t include the crown part, which could cause some slight differences in the difficulty of treating and administering the peri-implantitis with that of the actual clinical practice. Besides, there are usually at least 3 months for patients to achieve good osseointegration after the dental implant surgery. However, in our study, the induction of inflammation was conducted just 1 month after the surgery, which might become a contributor to the inflammation. In the following study, we will conduct research verification in clinical practice.

## Conclusions

This study demonstrated that the drug Mino-ZnO@Alb NPs has a better effect on treating patients with peri-implantitis than the traditional drug Minocycline. It can eliminate inflammation around the implant, restore soft tissue health, and inhibit the progress of bone resorption around the implant. This study will provide new therapeutic ideas for the treatment of peri-implantitis.
